# Evaluation of Genetic Pattern of Gentamicin-resistant Enterococci Isolated from Clinical Samples

**DOI:** 10.5681/joddd.2011.005

**Published:** 2011-03-18

**Authors:** Esrafil Balaei Gajan, Parisa Akbarzadeh, Bahram Harasi, Zohreh Moosavi

**Affiliations:** ^1^ Dentist, Department of Community Dentistry, Faculty of Dentistry, Tabriz University of Medical Sciences, Tabriz, Iran; ^2^ Assistant Professor, Department of Community Dentistry, Faculty of Dentistry, Tabriz University of Medical Sciences, Tabriz, Iran; ^3^ Post-graduate Student, Department of Oral and Maxillofacial Pathology, Faculty of Dentistry, Tabriz University of Medical Sciences, Tabriz, Iran; ^4^ Microbiology Laboratory, Faculty of Dentistry, Tabriz University of Medical Sciences, Tabriz, Iran

**Keywords:** Antibiogram, enterococcus, gentamicin

## Abstract

**Background and aims:**

Enterococci are among resistant microorganisms to antibiotics and are responsible for some of acute gingival infections. This study aimed at molecular evaluation of gentamicin-resistant enterococcus species and determining the resistance genes.

**Materials and methods:**

Clinical samples were cultured on BHI medium and enterococci were isolated by specific methods and antibiograms were prepared. DNA was derived from gentamicin-resistant species by alkaline lysis method and replicated by PCR method. Four gentamicin-resistant genes were evaluated by electrophoresis method on agarose gel.

**Results:**

From 105 *Enterococcus faecalis* isolates, 36.2% and from 38* Enterococcus faecium* isolates, 78.2% were resistant to gentamicin. 60% of these species bore aac (6’)- le – aph(2”)- la gene. ph(2”)- Ib,Ic,Id genes were not seen in any of the evaluated species.

**Conclusion:**

In gentamicin-resistant species of *E. faecalis* and *E. faecium,* the aac (6’)- le – aph(2”)- la gene was identified as the main cause of resistance.

## Introduction

Resistance to antibiotics is one of the critical complications in the treatment of infectious diseases. Enterococcal resistance to gentamicin, which is the effective antibiotic in this case, highlights the importance and the priority of the matter.^1-4^ Enterococci are among the most common microorganisms responsible for nosocomial infections in the hospital intensive care units and also the causative infectious agent in urinary and bile tract systems, resulting in bacteremia in infants and acute endocarditis in adults, in addition to dental and gingival infections.^5-8^, Therefore, isolation and identification of different enterococcal species and determination of their resistance pattern and their prevalence as well as obtaining their genetic patterns are of utmost importance.^7-10^ Examples of these methods include antibiogram by disk diffusion agar for determination of gentamicin resistance, evaluation of the genetic pattern utilizing DNA isolation, and observation of formed bands using electrophoresis on agarose gel 0.8% by ultraviolet.^8-11^, A general and comprehensive method for obtaining an ideal outcome by these methods can also be tested.

Enterococci are gram-positive cocci, usually detected in feces. These bacteria grow in blood agar or BHI containing 5% blood, and are often non-hemolytic but sometimes α- or β-hemolytic. They do not produce any gases and are PYR, LAP, and bile escolin positive. They grow in 6.5% NaCl medium and can bear the 10-45°C temperature range.^11-13^, The Moller Hinton medium is utilized for testing enterococcal resistance against gentamicin. In previous studies, the bacteria from diluted culture in 0.5 Mc Farland solution was cultured in Moller Hinton medium and the 100 µg gentamicin was placed on it and after 24 hours the resistance degree was reported by measuring the hallow around the disk.^3,7,8,13^ For determination of enterococcal resistance genetic pattern, the molecular and DNA derivation was used afterwards.

For plasmid derivation in alkaline lysis method, plasmids are turned to deluxe rapidly and remain soluble. Genomic DNA, however, can not do this because it is big and remains insoluble and sediments. The buffer’s potassium ions are combined with SDS molecules and produce the insolvable salt that causes the sedimentation of bonded polypeptides.^4,7,9^ After the complete cooling, for insulation of plasmids from buffer sediments’ derivations, the solution is centrifuged at high rpm and the surface solution is taken. Then for removing the salts and repurification, the phenol, chloroform, isoamil alcohol, andethanol were used and in the final stage, TE and distilled water were used for sample reservation.^9-11^

Generally, different stages of all DNA derivation methods lead to electrophoresis. Electrophoresis on agarose gel is a standard method for DNA isolation, recognition, and purification.^9,13^ The precise conclusion of electrophoresis is performed using different control tests. In most cases, the detection of one DNA band is of no importance and the comparison with control bands reveals its nature. So the design of suitable control tests is of utmost importance. For proper detection of each band at least 200-300 nanograms is required. Bands containing more than 500 nanograms DNA are less distinct or cause disturbance in electrophoresis.^9^ For electrophoresis of the derivate DNA on agarose gel, the DNA sample as well as the size marker with distinct DNA properties is electrophoresed, and then with comparing the samples’ band and the band for size marker, the formed DNA bands are evaluated. Size marker or plasmid pattern is the basis of species recognition with regards to the number and size of plasmids in a species.^1,7,9^ For this purpose, 10-15 µL of DNA solution with 5 µL of loading buffer are placed on 0.8% agarose gel and electrophoresis is carried out. After performing electrophoresis, the gel is stained by itidium bromide (0.5 µg/ml). UV transluminator is utilized for detection of DNA bands and then the bands are evaluated and analyzed.^7-9^

The aim of this was molecular evaluation of gentamicin-resistant enterococcus species and determining the resistance genes.

## Materials and Methods

The samples were cultured on blood agar or BHI agar and then the shape and gram-type of bacteria was determined by gram-staining. Growth in blood agar medium also enables the evaluating the hemolysis. Enterococci can be α- or β-hemolytic. In gram positive species, the isolation from staphylococci was carried out by catalase test. The 6.5% NaCl and pYR medium were used to isolate enterococci from group D streptococci and the enterococcal strain detection was performed by sugar fermentation. Then, the antibiogram was performed for determining gentamycin-resistant enterococci.^7-9,13^ Antibiogram requires Moller Hinton medium and gentamycin disks (100 mg).

### Antibiogram method

Colonies from 8-24 hours microorganism culture on BHI or blood agar was taken by a loop and transmitted to a glass containing sterile physiologic serum. The suspension’s turbidity was compared with 0.5 Mc Farland solution and the sterile physiologic serum was added if the turbidity was high. This sterile suspension should be used in a short time. A sterile cotton swab was soaked in microbial solution and excess water was taken by pressing it to glass wall. The swab was cultured on the Moller Hinton agar plate in a 360° motion in order to spread it homogenously. The plate lid was closed and the plate was placed in room temperature for drying the agar surface. Disks should not move on the medium surface. Then, the plate was incubated for 18-24 hours in 37°C. After 24 hours, plates were evaluated and the diameter of the area without growth was measured by a millimeter ruler and reported as ‘sensitive,’ ‘resistant,’ and ‘moderate.’^9,13^

### Plasmid DNA derivation

This procedure requires the following:^1,4,9,11,13^

1. Fresh Enterococcal culture on blood agar or BHI agar medium.
2. TE buffer solution (tris-EDTA) (pH=8) 10 mM tris HCl, 1 mM EDTA
3. Solution No. 1: TE buffer containing 25% sucrose and 10 mg/ml lysozyme. For the preparation of solution No. 1, 25 grams sucrose was dissolved in TE buffer and the final volume reached to 100 milliliters. The solution was kept in laboratory temperature and lysozyme powder that was kept at −20°C, was added to solution before use.
4. Solution No. 2: 0.2 mol NaOH, 1% sodium dodecyl sulfate (SDS). For the preparation of solution No. 2, 1 mL of 0.2 mol NaOH reserved solution was added to 8 mL of distilled water, then 1 mL of SDS 10% was added to it (The reserved 10% SDS solution was kept in the laboratory setting).
5. Solution No. 3: 3 molar potassium acetate (pH=4.8) kept at 4°C.
6. Phenol-chloroform, isoamil alcohol (with 1:24:25 ratio) kept in a dark bottle at 4°C.
7. Absolute alcohol (ethanol) kept at −20°C.

First, 100 µl of solution 1 was poured in centrifuge glasses and then enterococcus, cultured on BHI agar containing 5% sheep defibrinated blood, was taken by a standard loop and dissolved in it and incubated at 37°C for 30 minutes.

Then, 200 µl of solution 2 was added to it and, after mixing, was incubated at 56°C for 1 hour. After this time, 150 µl of cooled solution 3 (kept at 4°C in fridge) was added to it and mixed severely and centrifuged at high rpm (6000-8000 rpm) for 15 minutes. Then, the surface solution was taken by sampler and poured in a glass. After that, 400 µl of phenol, chloroform, and isoamil alcohol combination (with 1:24:25 ratio) was added to it and after mixing well, it was centrifuged at high rpm. At the next stage, 200 µl of surface solution was taken and transferred to clean microcentrifuge glasses. Then, 400 µl of cold ethanol was added and mixed and kept at room temperature for 2 minutes.^9,7,10,12^

### Electrophoresis

First, 54 grams of Tris base was dissolved in 800 ml of distilled water, then 3.72 gr EDTA was added to it. Finally, 27.83 grburic acid was dissolved in it and the final volume reached to 1 liter. Then, pH was tested for being alkaline. This solution was diluted 10 times by distilled water before use to reach 0.5× viscosity, capable of being used in electrophoresis tank.

- For the preparation of loading buffer, 50 landaubromophenol blue is combined with 3 cc glycerol and water.
- In order to prepare 0.8% agarose gel, regarding the required gel dimensions (the tank dimensions: 0.5×10×7.5 cm), 0.32 g of agarose was added to 2 cc of TBE X10 buffer and the volume reached to 40 mLby deionized distilled water (DDW). Then, it was boiled to be completely dissolved and clear. After cooling to 50°C, it was poured to electrophoresis plate containing comb and waited until fixation. After approximately half an hour, the comb was removed from gel and placed in tank and the TBE buffer was poured on it until the comb was floating. Then, the sample was loaded in it.

5 µl loading buffer was mixed well with 10 µl of DNA and then poured in pits. 0.2 µl of size marker is also poured in pits without loading buffer.

The electrophoresis was run at 90-100 volutes (10 volutes for each centimeter). The DNA moving direction was towards cathode side (positive pole) because of its inert load at inert pH. After removing gel, it was stained by itidium bromide for 10 minutes and then scanned in transluminator machine at camera range and then evaluated (the machine adjustment was on UV). UV wave caused DNA bands indication during transmitting through gel.^1-4,7-9,12,13^

## Results

From 139 Enterococcal isolates, 105 and 34 instances were Enterococcus faecalis and Enterococcus faecium, respectively.

Among 105 enterococcus faecium isolates, 38 (33.4%) and among 34 enterococcus faecium isolates, 17 (50%) were gentamicin-resistant.

In HLGR isolated enterococci, four resistance genes were evaluated with these primers:

- Aac(6)-Ie-aph-(2)-Ia (369 bp)
- Aph(2)-Ib (867 bp)
- Aph(2)-Ic (444 bp)
- Aph(2)-Id (307 bp)

Among 38 gentamicin-resistant Enterococcus faecalis samples, 32 (84.4%) instances bore Aac(6)-Ie-aph-(2)-Ia gene. Aph(2)-Ib-c-d genes were not detected in any of the evaluated species.

Among 17 gentamicin-resistant Enterococcus faecium samples, 11 (64.7%) instances bore Aac(6)-Ie-aph-(2)-Ia gene. Aph(2)-Ib-c-d genes were not detected in any of the evaluated species.

## Discussion

The presence of antibiotic resistance phenotypes in bacteria is indebted to plasmids and their high potential for numerous mutations, leading to the development of new genes. Each cell can only endure one type of plasmid in its cytosol, and in the elevation of the plasmid type, the cell is divided to two daughter cells, regarding Mandel principles.

Plasmids are naturally circular chromosomes without any small proteins, and are seen in bacteria as well as the bacterial genome. Their genome is composed of double-stranded DNA and their replication is dependent on the host cells but independent of the bacterial genome. Most of them are replicated in bacterial reproduction cycles. Plasmids bear many genes that the bacterium usually needs their productions and for this, their high quantities are justifiable.

For plasmid derivation in alkaline lysis method, plasmids are turned to deluxe rapidly. Generally, when the antibiotic concentration required for bacterial death or its growth inhibition is higher, the microorganism shows resistant to it. Previously aac(6)-Ie-aph(2)-Ia gene was presumed responsible for gentamicin resistance. This gene is carried by plasmids and is located on Tn Ann I staphylococcal transposon. This transposon has a central part that is the location of gene with the insertion sequence (I S 256) on each side. In recent years, three other genes including aph(2)-Ic, aph(2)-Id, and aph(2)-Ib have been identified that cause gentamicin resistance.

Among 55 gentamicin-resistant enterococcus samples, 43 instances bore Aac(6)-Ie-aph-(2)-Ia gene that indicates its crucial role in resistance to gentamicin in enterococci. It seems Aph(2)-Ib-c-d genes do not play an important role in gentamicin resistance among enterococci. In the case of HLGR enterococci, the molecular assessment of isolated spices and resistance determination reveal useful information for detection of the pattern of resistance gene transmission among spices and also simplifies the problem diagnosis and antibiotic treatment of enterococci infections.
